# On the Curative Medication of Intermittent Fever

**Published:** 1846-11

**Authors:** 


*On the Curative Medication of Intermittent Fever.* By M. Bretonneau.—A sort of drunkenness, more or less painful, produced by a single and suitable dose of sulphate of quinine, repeated, if necessary, two days afterwards, suppresses for eight days simple intermittent fever. In the same way that with a person who is in the habit of intoxication, we find that much wine may be taken without producing this condition; so we every day see individuals affected with ague taking great quantities of quinine, without the fever becoming suppressed, or its return being prevented. M. Bretonneau agrees with the Jesuits who first imported cinchona, and learned how to administer it; and with Tarti, who for thirty years practised in the hospital at Tours, that every sufficient dose of bark loses its febrifuge power by fractioning it, exactly as a dose of wine loses its intoxicating property by being divided.

It has been stated that the prolonged administration of many doses, whose sum altogether amounted to several efficient doses, has completely failed. M. Bretonneau has seen a quartan fever, which had resisted two ounces of bark, yield to two drachms of the same medicine; but the two drachms were given at once, while the two ounces were taken in very small quantities at a time, and during fifteen days. Small doses, which habituate a patient to the action of cinchona, injure the beneficial results of adequate doses; they hurt the digestive apparatus, and render the febrifuge intoxication more difficult to obtain.

M. Bretonneau corroborates the statements of Sydenham and Morton—that the dose of bark which has suppressed a paroxysm, if repeated before the supposed epoch of its return, will prevent it; and moreover, that the immunity thus procured may be prolonged by renewed doses exhibited at gradually lengthened intervals, until a permanent immunity from relapse becomes established. The progression recommended in the giving of these preservative doses is the following:—a second dose equal to that which suppressed the fever, is to be exhibited according to the nature of the fever, on any day from the first to the sixth interval, then to be repeated after intervals of 7, 8, 9, 10, 12, 14, 16, 18, 22, 30, days. The best time for giving each preservative dose is immediately after a light dinner; and the first dose had better be given shortly after the decline of the suppressed access, so as to be as distant as possible from the next threatened paroxysm. A relapse will render it necessary to renew the treatment from the commencement. The preservative doses should be approximated if it is found that the accesses become more frequent.

From fifteen to twenty grains of the sulphate of quinine, or from three to four drachms of good cinchona, suffice to suppress the fever of an adult, and to keep it suppressed during eight or nine days. Many reasons lead to the belief that it is useful that the necessary dose should not be exceeded.

*Dublin Hosp. Gaz.—Braithwaite's Ret.*

